# Up-Regulation of SOX9 in Sertoli Cells from Testiculopathic Patients Accounts for Increasing Anti-Mullerian Hormone Expression via Impaired Androgen Receptor Signaling

**DOI:** 10.1371/journal.pone.0076303

**Published:** 2013-10-01

**Authors:** Kuo-Chung Lan, Yen-Ta Chen, Chawnshang Chang, Yung-Chiao Chang, Hsin-Jung Lin, Ko-En Huang, Hong-Yo Kang

**Affiliations:** 1 Department of Obstetrics and Gynecology, Kaohsiung Chang Gung Memorial Hospital and Chang Gung University College of Medicine, Kaohsiung, Taiwan; 2 Graduate Institute of Clinical Medical Sciences, Chang Gung University, Kaohsiung, Taiwan; 3 Hormone Research Center, Kaohsiung Chang Gung Memorial Hospital and Chang Gung University College of Medicine, Kaohsiung, Taiwan; 4 Department of Urology, Kaohsiung Chang Gung Memorial Hospital and Chang Gung University College of Medicine, Kaohsiung, Taiwan; 5 George H. Whipple Lab for Cancer Research, Departments of Pathology, Urology and Radiation Oncology, and the Wilmot Cancer Center, University of Rochester Medical Center, Rochester, New York, United States of America; Blaise Pascal University, France

## Abstract

**Background:**

Testosterone provokes Sertoli cell maturation and represses AMH production. In adult patients with Sertoli-cells-only syndrome (SCOS) and androgen insensitivity syndrome (AIS), high level of AMH expression is detected in Sertoli cells due to defect of androgen/AR signaling.

**Objective:**

We postulated that up-regulation of SOX9 due to impairment of androgen/AR signaling in Sertoli cells might explain why high level of anti-Mullerian hormone (AMH) expression occur in these testiculopathic patients.

**Methods:**

Biological research of testicular specimens from men with azoospermia or mouse. The serum hormone levels were studied in 23 men with obstructive azoospermia, 33 men with SCOS azoospermia and 21 volunteers with normal seminograms during a period of 4 years.

Immunohistochemical staining and reverse-transcription PCR were used to examine the relationships among AR, SOX9 and AMH expression in adult human and mouse testes. The ability of AR to repress the expression of SOX9 and AMH was evaluated *in vitro* in TM4 Sertoli cells and C3H10T1/2 cells.

**Results:**

SCOS specimens showed up-regulation of SOX9 and AMH proteins but down-regulation of AR proteins in Sertoli cells. The mRNA levels of AR were significantly lower and the SOX9, AMH mRNA levels higher in all SCOS patients compared to controls (*P*< 0.05). The testosterone levels in the SCOS patients were within the normal range, but most were below the median of the controls. Furthermore, our *in* *vitro* cell line experiments demonstrated that androgen/AR signaling suppressed the gene and protein levels of AMH via repression of SOX9.

**Conclusions:**

Our data show that the functional androgen/AR signaling to repress SOX9 and AMH expression is essential for Sertoli cell maturation. Impairment of androgen/AR signaling promotes SOX9-mediated AMH production, accounts for impairments of Sertoli cells in SCOS azoospermic patients.

## Introduction

Androgen and the androgen receptor (AR) have been shown to play critical roles in testis function [1-3]. The Sertoli cells of the testes play a crucial supportive/nursing role throughout germ cell differentiation. However, mice with AR-deficient Sertoli cells showed altered testosterone production, changes in the secretion function of Leydig cells and impaired spermatogenesis, resulting in azoospermia and infertility [4-7]

Sertoli cells secrete anti-Mullerian hormone (AMH) [8,9], a transforming growth factor-like hormone that causes regression of the Mullerian ducts during the embryonic development of gonads. Immunohistochemical staining of AMH in testicular biopsies from fetal, neonatal, prepubertal, pubertal, and adult human testes showed that AMH immunolabeling was strong in all Sertoli cells from fetal life throughout prepuberty, and then weakened progressively as spermatogenesis developed [10]. The serum levels of AMH and testosterone are negatively correlated during puberty and adulthood [11], indicating that testosterone could be responsible for inhibiting AMH production in Sertoli cells. Recently, a lack of AR expression in Sertoli cells was found to account for the absence of AMH repression during early human testicular development [12]. However, the mechanisms that allow androgen/AR signaling to halt AMH expression are not yet known.

From a molecular viewpoint, it has been demonstrated that AMH is a downstream target gene of SOX9, which is a member of the SOX [Sry-related high-mobility group (HMG) box] family. SOX9 interacts with steroidogenic factor 1 on the AMH promoter to directly stimulate AMH expression [13,14], thereby playing a critical role for male sex determination in the developing gonad [15-17]. SOX9 protein is distinctly expressed in developing and mature Sertoli cells, where its expression and function depend on age and the stage of spermatogenesis within the seminiferous tubule [18]. However, although both animal and cell line data have demonstrated that SOX9 plays a critical role in testicular determination [19,20], the physiological relevance and pathological roles of SOX9 in adult human testes warrant further investigation.

In testicular biopsy specimens, AMH immunoreactivity is seen in the immature Sertoli cells of the normal postnatal testis, but gradually disappears in adult testis undergoing normal spermatogenesis after puberty [12]. Notably, however, high-level AMH expression can be detected in the immature Sertoli cells of adult patients with Sertoli-cell-only syndrome (SCOS) or AIS [12,21-23]. The AIS is the most frequent infertility condition among the steroid hormone resistance syndromes [24]. Affected individuals have a 46,XY karyotype and testes, but show a spectrum of hypovirilization, such as infertility secondary to azoospermia and oligospermia, reduced pubertal virilization with normal male genitalia (mild AIS), and individuals with a female genital phenotype (complete AIS). SCOS (mild AIS) is one of the most frequent pathological pictures characterizing complete absence of spermatozoa [25,26]. Here, we sought to test the hypothesis that the deficiency of spermatogenesis in testiculopathic testes is related to the down-regulation of androgen/AR signaling and the subsequent up-regulations of AMH and SOX9 in adult testicular Sertoli cells. We used immunohistochemistry and real-time quantitative RT-PCR to compare the mRNA and protein expression profiles of AR, SOX9 and AMH in testes from patients and mice with normal and deficient spermatogenesis. In addition, we used an *in vitro* model to demonstrate the reciprocal relationship between AR and SOX9.

## Materials and Methods

### Patients and tissue collection

Male patients with obstructive and non-obstructive azoospermia were recruited from infertility clinics at the Chang Gung Memorial Hospital in Kaohsiung during office visits for testicular sperm extraction and assisted reproductive therapy. The urological services of the hospital counseled the patients to undergo testicular sperm extraction (TESE) using the previously described protocol [27]. The collected testicular samples were immediately sent for pathological examination, and subsamples were stored in liquid nitrogen for RNA extraction. Patients who had histological confirmation of normal or deficient spermatogenesis (maturation arrest, germ-cell aplasia, and tubular sclerosis/atrophy) were collected. Patients with hypospermatogenesis, chromosomal anomalies or cryptorchidism were excluded, as we wanted to compare a well-defined group with testicular failure versus patients with normal spermatogenesis. Two distinct histological patterns were distinguished by immunostaining of the seminiferous tubules: 1) tubules containing mature germ cells (normal spermatogenesis), and 2) tubules that had germ cell aplasia and Sertoli cells only (deficient spermatogenesis). The diagnosis of SCOS was established if only tubules with Sertoli-cells were detected in bilateral and multilocular (at least two sites pertestis) testicular biopsies and the attempt of testicular sperm extraction (TESE) was unsuccessful [25,26].

### Ethics statement

This study was approved by the Ethics Committee of Chang Gung Memorial Hospital. Approval from the institutional review board was obtained for the analysis of this series

(CGMH97-2399A3). All participants provided their written informed consent to participate in this study. All animal procedures followed the ***GuidefortheCareandUseofLaboratoryAnimals*** as promulgated by the Institute of Laboratory Animal Resources, National Research Council, National Academy of Science (United States), and were approved by the Animal Care and Use Committee of the Chang Gung Memorial Hospital at Kaohsiung Medical Center.

### Immunohistochemical and immunofluororescence staining

Tissues were paraffin-embedded and subjected to immunohistochemical and immunofluororescence staining. The sections were deparaffinized, rehydration, retrieval with 10 mM citrate buffer and then incubated with primary antibodies. The AR (N-20, Santa Cruz, CA, USA), SOX9 (N-17, Santa Cruz) and AMH (AF2748, R&D Systems, Minneapolis, MN, USA) antibodies were diluted 1:100, 1:50 and 1:50, respectively. After the appropriate secondary IgG antibody, the sections were incubated with DAB (Dako, Glostrup, Denmark) and counterstained with Gill’s hematoxylin (Merck, Whitehouse, NJ, USA). The integrated pixel intensity was determined for the traced areas using the Image-pro Plus image analysis software (Media Cybernetics, Silver Springs, MD, USA).

For double immunofluororescence staining with AR and SOX9 antibodies, the sections were incubated overnight with AR (Santa Cruz) and SOX9 (AB5535, Millipore) in a humidified chamber at 4°C after the microwave antigen retrieval and treating with 10% horse serum to blocking any nonspecific antibody binding. AR and SOX9 antibody binding was visualized using ***AlexaFluor*** ^®^ ***488*** Goat Anti-Rabbit ***IgG*** or ***AlexaFluor*** ^®^ ***568*** Goat Anti-Rabbit ***IgG*** (H+L) (Invitrogen). Finally, the sections were counterstained with DAPI (Santa Cruz) and mounted in a 50% glycerol solution. All samples were visualized under the fluorescence microscope (Nikon E800, Melville, NY) and the images were captured and processed by a SPOT RT3 digital camera and software (Diagnostic Instruments, MI).

### RNA Extraction and Quantitative real-time reverse transcriptase-polymerase chain reaction (RT-PCR)

Total RNA was isolated using TRIzol reagent (Invitrogen Inc., Carslbad, CA, USA) and reverse transcribed. Real-time reverse transcription-PCR (RT-PCR) was performed by using a Sybr green PCR master mix kit (PE Applied Biosystems, Foster City, USA) as previously described [27,28]. Sequence analysis was performed by using an ABI Prism 7700 sequence detection system (PE Applied Biosystems). The sequences of all primers used in the present study are listed in Table S1 in the supplemental material. We evaluated the value and reliability of six potential reference genes (18S rRNA, GAPDH, β-actin, B2M, HPRT1 and TBP) for real-time PCR in the testis tissue. In agreement with previous study [29] by using geNorm bioinformatic tool, 18S and GAPDH were identified as the most stable housekeeping genes in testis groups. We also applied REST 2009 Software [30], a mathematic model that takes into account the different PCR efficiencies of the gene of interest and reference genes. Relative quantification was performed using average of 18S and GAPDH as internal controls

### Serum hormone assays

Azoospermic men (23 and 33 patients with obstructive and SCOS azoospermia, respectively) were enrolled and detailed chart reviews were conducted. As part of the routine male azoospermic infertility workup in our institute, the serum hormone levels of luteinizing hormone (LH), follicular stimulating hormone (FSH), and testosterone (T) were assessed by commercially available RIAs (Yatoron, Tokyo, Japan). The detection limits were 0.3 mIU/ml for FSH, 0.1 mIU/ml for LH, and 0.05 ng/ml for testosterone. Twenty-one volunteers with normal seminograms were studied as controls.

### Animals, cells culture, plasmids, and transfection

We were using Cre-Lox strategy to generate WT and ARKO mice [28]. WT and ARKO mice were established by mating floxed AR/AR female mice with ACTB- Cre mice. The ARKO mice were genotyped by PCR as described previously [28]. Animals were housed in pathogen-free facilities, maintained on a 12-h light/dark schedule (light on at 6 a.m.).

The C3H10T1/2 cell and TM4 cell line were obtained from the American Type Culture Collection (ATCC). C3H10T1/2 cells, a cell line established from mouse embryonic connective tissue, were cultured at in DMEM (Invitrogen, Carslbad, CA, USA) containing 10% fetal bovine serum (FBS, Invitrogen), 100 U/mL penicillin/streptomycin (Invitrogen) and 2 mM L-glutamine (Invitrogen). The male mouse Sertoli TM4 cells were maintained in DMEM/F12 (Invitrogen) supplemented with 2.5% FBS, 5% horse serum (HyClone, Logan, Utah, USA), 100 U/mL penicillin/streptomycin, and 2 mM L-glutamine. All cells were grown in a 5% CO_2_ humidified atmosphere at 37°C.

The AR expression vector (pcDNA3-flag-AR) and its control (pcDNA3-flag) were performed as previous described [28]. The Mouse AR and SOX9 RNAi used in this study were obtained from the National RNAi Core Facility (Institute of Molecular Biology, Academia Sinica, Taipei, Taiwan). Cells were plated at 5×10^4^ cells/well in six-well plates (BD Falcon Labware, Franklin Lakes, NJ, USA), or at 5×10^5^ cells in 100-mm culture dishes, and incubated for 24 h. Transiently transfection were using the Lipofectamine 2000 reagent (Invitrogen) according to the manufacturer’s instructions. After 12-16 h, the cells were transferred to medium supplemented with charcoal-dextran-treated serum, and then treated with EtOH or DHT (1 x 10^–8^ M) or flutamide (10µM) (F9397, Sigma), an androgen antagonist, for 24-48 h.

### Western blot analysis

Cells were lysed in RIPA buffer, resolved by 8~10% SDS/PAGE, and then transferred to nitrocellulose membranes (Amersham Biosciences, Piscataway, NJ, USA). The membranes were probed with the appropriate primary antibody and then developed using an ECL kit (Millipore, Billerica, MA, USA). The utilized primary antibodies included anti-AR (PG21, Millipore), anti-SOX9 (AB5535, Chemicon, Billerica, MA, USA), anti-MIS (PP07, Santa Cruz), anti-GAPDH (MAB374, Millipore), and anti-β-tubulin (D-10, Santa Cruz).

### Statistical analysis

The SPSS 10.0 computer software package (SPSS, Inc., Chicago, IL, USA) was used for data analysis. Continuous data were summarized as the mean ± standard deviation (SD). All *P* values are two-sided, and a *P* < 0.05 was considered statistically significant. The non-parametric Mann-Whitney U test was used to evaluate expression differences between patients and controls.

## Results

### Immunohistochemical and RT-PCR analysis of AR, SOX9 and AMH in azoospermic testes

**Figure 1:**
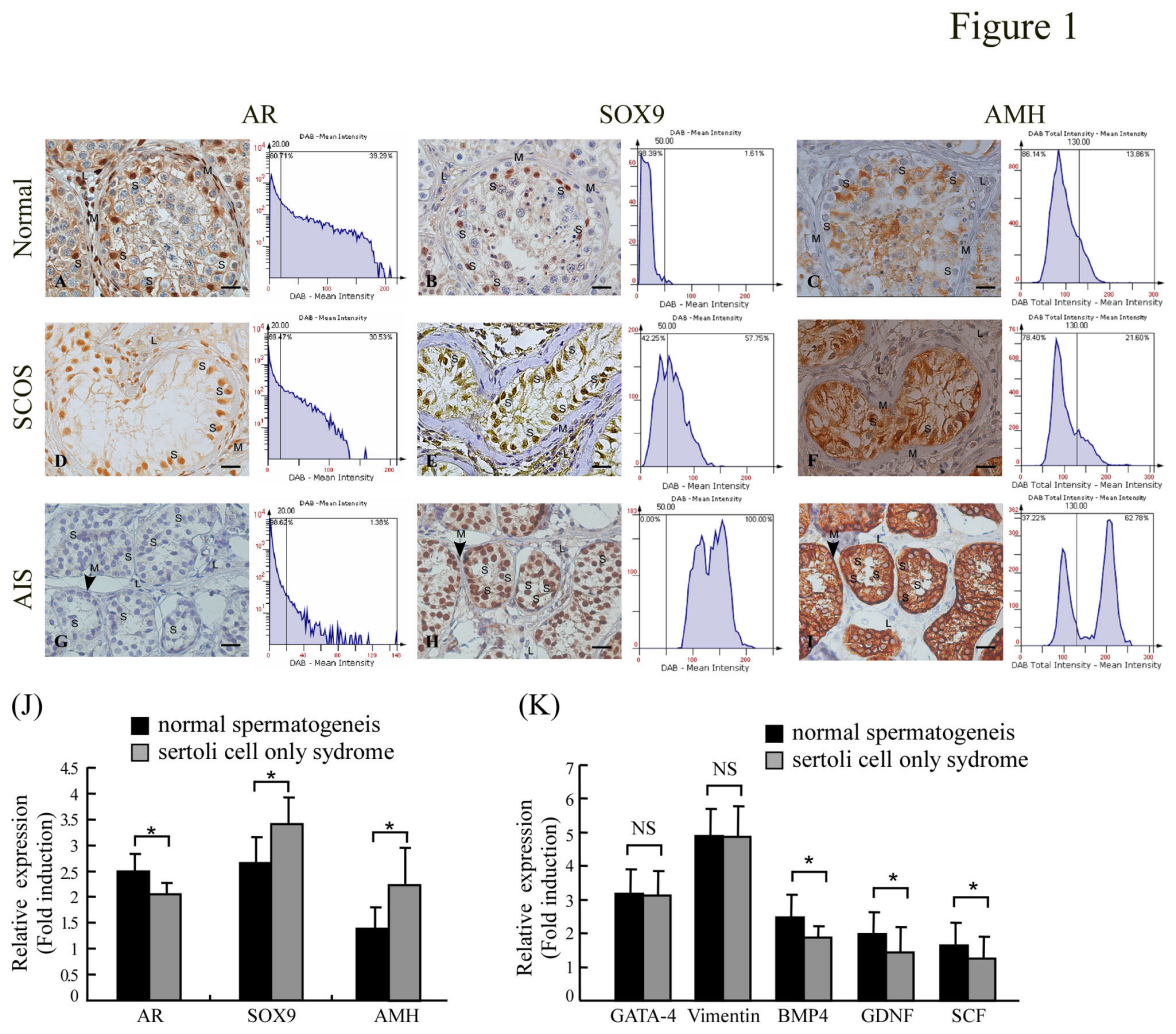
Comparative analysis of AR, SOX9 and AMH expression in adult human testes representing androgen-insensitivity syndrome (AIS), Sertoli-cell-only syndrome (SCOS), and normal spermatogenesis. The localization and expression of AR, SOX9, and AMH proteins were analyzed by immunohistochemical analysis. (**A**, **D**, **G**) AR in normal, SCOS and AIS testes. (**B**, **E**, **H**) SOX9 in normal, SCOS and AIS testes. (**C**, **F**, **I**) AMH in normal, SCOS and AIS testes. No AR expression was detected in AIS testes. Note that all Sertoli (S) and peritubular myoid (M) cell nuclei showed AR expression in SCOS and normal samples. Leydig (L) cells with AR-positive nuclei were not consistently detected in SCOS. SOX9 expression was predominantly observed in the Sertoli cells. AMH was strongly expressed in the Sertoli cell cytoplasm of AIS and SCOS testes. The corresponding histograms show the mean relative intensities of DAB in the human testes biopsies. Automated acquisition and tissue cytometric cell measurement analysis were performed using the TissueFAXS system (TissueGnostics, Vienna, Austria) and HistoQuest (TissueGnostics) Analysis Software. There was an inverse correlation between the immunostaining density of AR and those of SOX9 and AMH in human testes. Magnification ×400; bar =20 µm. (**J**) The AR, SOX9, AMH and (**K**) several sertoli cell markers gene transcripts in testicular tissues from men between control and SCOS group were analyzed by real-time RT-PCR. The mRNA expression of SOX9 and AMH gene had an increasing trend, but AR gene transcript had a decreasing trend in the SCOS group compared to the normal spermatogenesis group. All data are representative of at least three independent experiments and each bar represents mean ± SD. Concomitant detection of 18S/ GAPDH mRNA served as a reference for relative quantification. *P <0.05.

**Figure 2:**
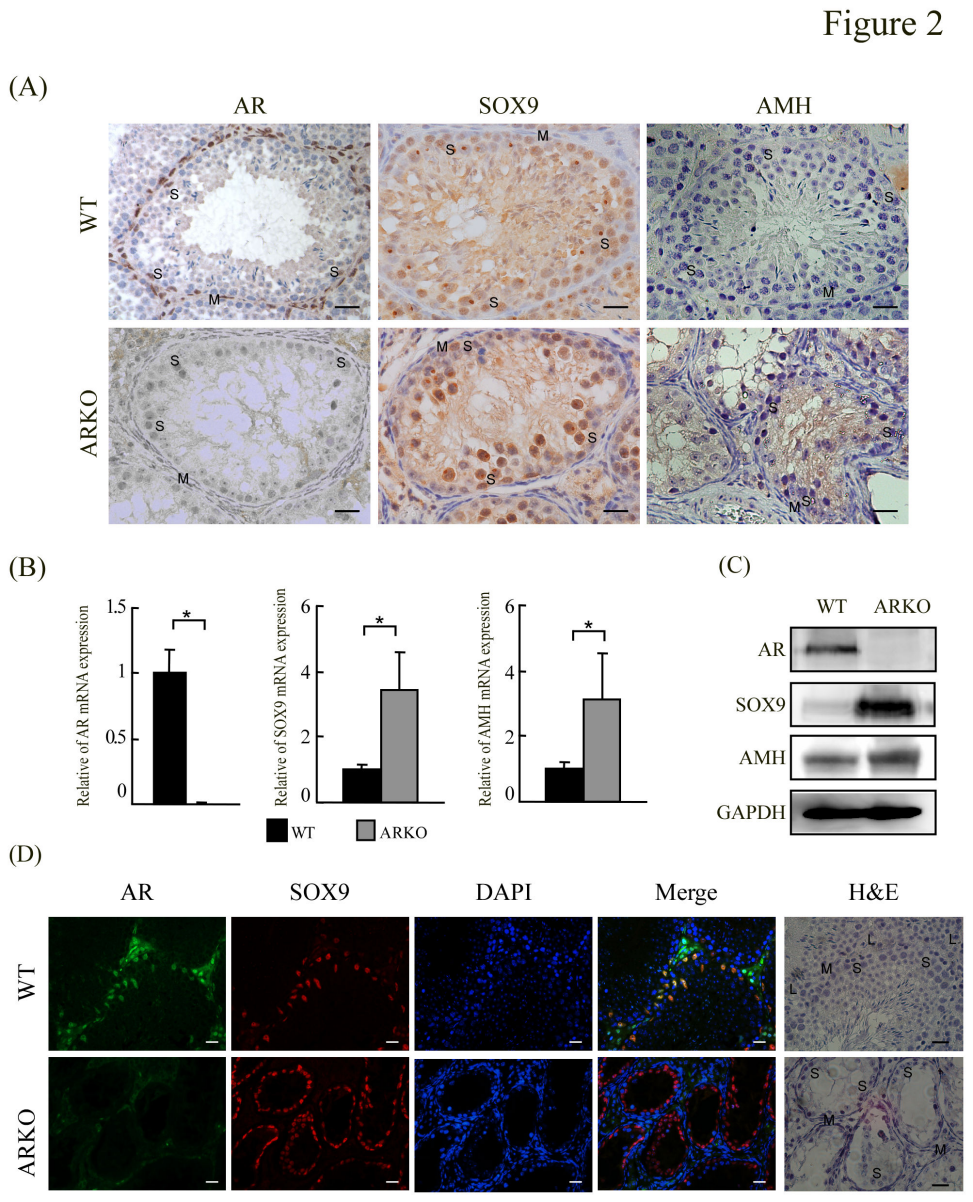
Immunohistochemical analysis of mouse testes. (**A**) AR, SOX9 and AMH immunostaining of testes obtained from wild-type and AR knockout (bottom panel) mice. Note that all Sertoli (S) cell nuclei, peritubular myoid (M) cell nuclei and Leydig (L) cells were AR-positive in wild-type mouse testes. Sertoli (S) cell nuclei showing SOX9 expression and Sertoli (S) cell cytoplasm with weaker AMH immunostaining for a wild-type mouse are shown in the upper panel. Sertoli (S) cell nuclei showing SOX9 distribution in an AR knockout mouse. Specific and robust AMH immunostaining was detected exclusively in the cytoplasm of Sertoli (S) cells in AR-knockout mouse testes with deficient spermatogenesis. Magnification ×400; bar = 20 µm. (**B**) Quantitative evaluation of AR ,SOX9 AMH in testicular tissues from wild-type and AR-knockout mice by real time RT-PCR. Each bar represents the mean ± SD; results were normalized with respect to 18S/GAPDH mRNA expression. SOX9 and AMH mRNA expression was significantly higher in testes from AR-knockout mice compared to controls. (C) SOX9 and AMH protein expression was significantly higher in testes from AR-knockout mice compared to controls by Western blot. (**D**) Co-immunostaining for AR and SOX9 Sertoli (S) cell nuclei of wild-type mouse testis. Magnification ×400. bar = 20 µm.

To examine whether up-regulations of AMH in testiculopathic testes with the deficiency of spermatogenesis is related to the expression of SOX9 and AR. Immunohistochemical analysis was used to investigate the expression of AR, SOX9 and AMH in SCOS azoospermic and control (normal and AIS) testes (Figure 1). Consistent with previous reports [2,27,31], specific AR immunostaining was detected in the nuclei of Sertoli, Leydig, and peritubular myoid cells of adult human testis with normal spermatogenesis, but not in the germ cells (Figure 1A). In SCOS patients, the majority of the Sertoli, Leydig and peritubular myoid cell nuclei of also exhibited AR immunostaining (Figure 1D), but AR immunostaining was absent from the bizarre seminiferous tubule architecture of AIS testes (Figure 1G). However, SOX9 proteins were exclusively detected in human testicular Sertoli cells nuclei (Figure 1B, E and H) and AMH proteins were specifically detected in the cytoplasm of Sertoli cells; neither signal was detected in Leydig or peritubular myoid cells (Figure 1C, F and I). Interestingly, the AIS biopsies showed significantly stronger SOX9 and AMH staining in Sertoli cells, plus an absence of AR staining (Figure 1; compare H and I vs. B and C). Stronger SOX9 and AMH staining plus weaker AR staining was also observed in SCOS patients (Figure 1; compared E and F vs. B and C). There were clear reverse correlations in the intensities of SOX9 versus AR staining, and those of AR versus AMH staining. To confirm the correlations among the expression levels of AR, SOX9 and AMH, the immunostaining and mRNA and protein expression levels of AR, SOX9 and AMH were examined in testes from male AR-knockout and control mice. Consistent with previous report [6], AR knockout mice germ cell development was severely disrupted, which was similar to testicular feminization mouse (Figure 2A bottom panel). SOX9 proteins were exclusively detected in mice testicular Sertoli cells nuclei. Similar to our observations in AIS patients, strong SOX9 and AMH staining was detected in the Sertoli cells of ARKO mice (Figure 2A), and both AR and SOX9 were expressed and co-localized in the nucleus of wild-type mouse Sertoli cells (Figure 2D). In the testes of ARKO mice, SOX9 and AMH mRNA and protein expression were significantly higher compared with the control mice (P < 0.05) (Figure 2B, 2C and Figure S1B).

We then used real-time RT-PCR to quantitatively analyze the expression levels of AR, SOX9 and AMH in the 23 and 33 patients with obstructive azoospermia with normal spermaotgensis and SCOS, respectively. In the testes of SCOS patients, SOX9 and AMH mRNA expression was significantly higher and AR mRNA expression was lower compared with the normal controls (P < 0.05) (Figure 1J and Figure S1A). We also found the mRNA levels of several sertoli cell markers (Table 1) were decreased or similar in SCOS patients compared to controls (Figure 1K). The serum levels of LH and FSH were higher in SCOS patients, and although their serum testosterone levels were within the normal range, most were below the median level of the controls (Table 1). Taken together, these results indicate that SOX9 and AMH are up-regulated and AR is down-regulated in the Sertoli cells of human testes with impaired spermatogenesis and abnormal seminiferous tubule structure.

**Table 1:** Hormone levels shown in three groups of men: control patients with normal sperm parameters (group 1), obstructive azoospermia with normal spermatogenesis (group 2) and SCOS (group 3).

	Men with normal sperm parameters		Obstructive azoospermia (normal spermatogenesis)	SCOS
No	21		23	33
Age (y/o)	35.1 ± 3.1		34.1 ± 4.4	35.5 ± 3.8
LH (mIU/mL)	3.8 ± 2.4		4.0 ± 2.1	11.0 ± 5.8**
FSH (mIU/mL)	5.8 ± 3.3		5.0 ± 3.1	25.5 ± 12.3**
Testosterone (ng/mL)	3.5 ± 0.9		3.8 ± 1.4	2.6 ± 0.9*

Note: Values are mean ± SD.

* *P* < 0.05

** *P* < 0.001

### Androgen/AR signaling negatively regulates Sox9 and AMH expression *in vitro*

**Figure 3:**
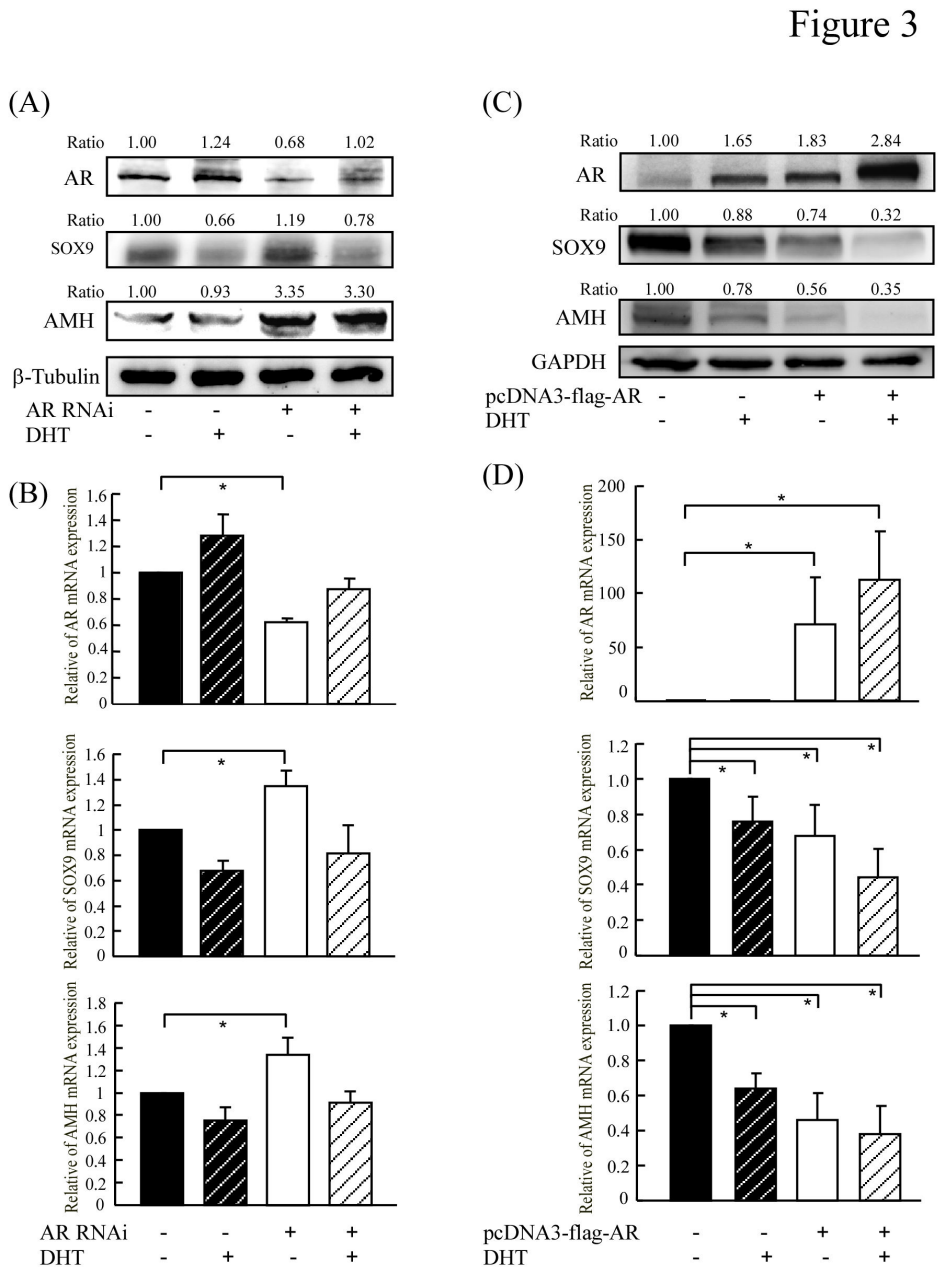
The expressions of SOX9 and AMH are repressed by androgen/AR. (**A** and **B**) TM4 cells transfected with AR RNAi and treated with or without DHT for 24~48 hours were analyzed by Western blot and quantitative real-time RT-PCR. (**C** and **D**) C3H10T1/2 cells transfected with pcDNA3-flag-AR plasmid, followed by incubation with or without 1x10^-8^ M DHT for 24~48 hours. The expression of AR, SOX9 and AMH were analyzed by Western blot and quantitative real-time RT-PCR. The optical densities obtained for protein and mRNA expression from vector with vehicle treatment were normalized using GAPDH,β-tubulin or β-actin expression levels and set as 1. All data are representative of at least three independent experiments and error bars represent ± SD. Asterisks (*) mark samples significantly different with P <0.05.

Next, we determined whether SOX9 and AMH were increased in response to down-regulation of androgen/AR expression in an *in vitro* cell culture model. TM4 cells (a mouse Sertoli cell line) were transfected with AR RNAi and treated with or without DHT (1x10^-8^M), and quantitative real-time RT-PCR was used to analyze the mRNA expression levels of SOX9 and AMH. We found that the protein and mRNA expression levels of SOX9 and AMH were suppressed by DHT in control cells but significantly increased in AR-knockdown cells (Figure 3A and B). We then used RT-PCR and Western blotting of cells transiently transfected with an AR-encoding expression vector to examine the effect of AR overexpression on the protein and mRNA levels of SOX9 and AMH. As shown in Figure 3C, the SOX9 and AMH protein levels in control cells were modestly reduced after DHT treatment, and this suppressive effect was enhanced by AR overexpression. Similar results were observed when we examined the mRNA expression levels of SOX9 and AMH (Figure 3D). We have checked SOX9 mRNA level by treating with flutamide along with androgen. The results showed that not only the AR expression level but also AR activity regulated by DHT or flutamide can affect the SOX9 expression level as showing in Figure S2.

### SOX9 is essential for androgen/AR signaling to down-regulate AMH expression *in vitro*

**Figure 4:**
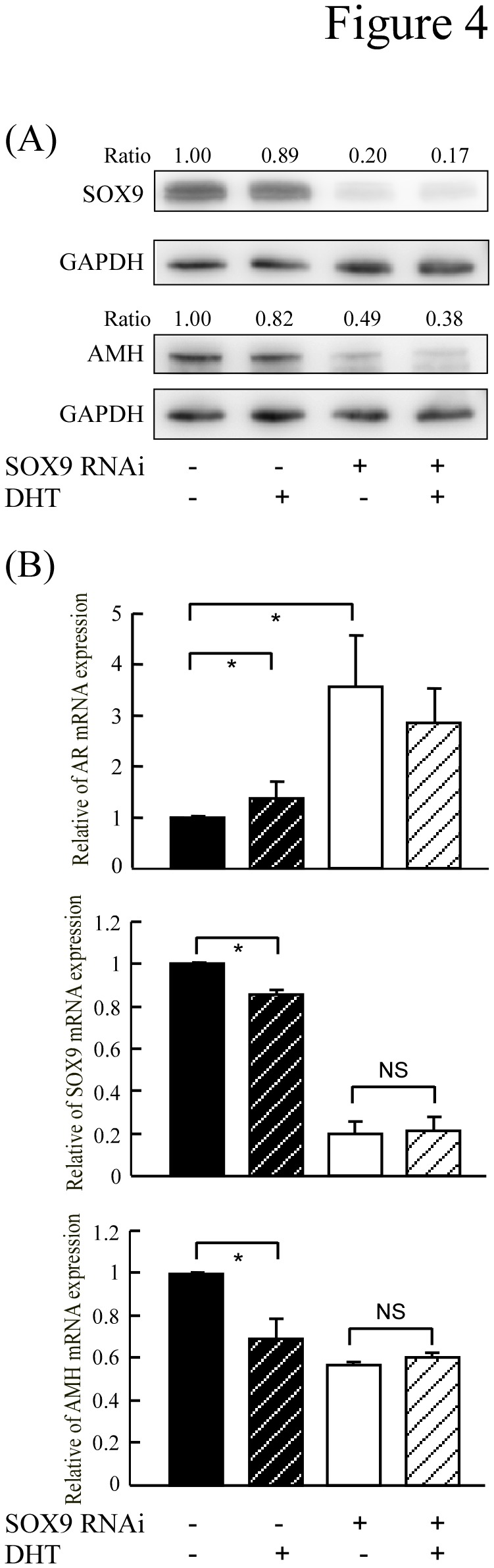
SOX9 is required for androgen/AR to repress AMH expression. C3H10T1/2 cells stably transfected with SOX9 RNAi and treated with or without DHT for 24~48 hours and the expression of SOX9 and AMH were analyzed by Western blot (**A**) and quantitative real-time RT-PCR (**B**). The optical densities obtained for protein and mRNA expression from vector with vehicle treatment were normalized using GAPDH or β-actin expression levels and set as 1. All data are representative of at least three independent experiments and error bars represent ± SD. Asterisks (*) mark samples significantly different with P <0.05.

It has been well documented that SOX9 plays a critical role in male sex determination by directly binding the AMH promoter to stimulate AMH expression in the developing gonad (11,12). To determine whether SOX9 is required for androgen/AR to decrease AMH expression, we stably transfected C3H10T1/2 cells with SOX9 RNAi and examined whether SOX9 knockdown affected the ability of androgen/AR to inhibit AMH expression. As shown in Figure 4, SOX9 knockdown significantly decreased AMH expression on both the protein and mRNA levels. Interestingly, although DHT decreased the levels of AMH mRNA in control cells, this inhibition was not seen in SOX9-knockdown cells.

## Discussion

In adult males, the action of testosterone on seminiferous tubules is essential for the full maturation of normal sperm, in a process known to be largely mediated by androgen/AR-mediated gene changes. However, the downstream mechanisms underlying the actions of AR on Sertoli cells are not yet fully understood. SOX9 is required to regulate several aspects of testicular development and function in mouse models [18,32,33]. The prominent presence of SOX9 in the incipient testis [34] suggests that this protein is needed for important phases of Sertoli cell aggregation during early testicular development. The age- and stage-specific presence of SOX9 in the testicular cords and seminiferous tubules suggests that SOX9 may play a pivotal role in germ cell differentiation [18]. However, the importance of SOX9 has not yet been examined in human testis, and we do not yet fully understand the physiological relevance of SOX9 in normal and pathological human testes. Here, we report for the first time that SOX9 is highly expressed in testiculopathic testes with impaired spermatogenesis from SCOS patients compared to human testes with normal spermatogenesis. Mechanistically, we show that decreased androgen/AR expression promotes the ability of SOX9 to activate AMH production in Sertoli cells.

In mammals, AMH gene expression is positively triggered by SOX9 in Sertoli cells at the onset of testicular differentiation, and regulated by SF1, WT1, DAX1 and FSH [35,36]. An interaction of SOX9 and SF1 was found to be essential for binding to the AMH gene promoter [13], and participated in the cyclic AMP-mediated up-regulation of AMH transcription in a testicular prepubertal Sertoli cell line [14]. Interesting, AR was shown to suppress the activity of the LHbeta promoter through protein-protein interactions with SF-1 [37], and overexpression of exogenous SOX9 decreased AR expression and activity in prostate cells [38]. As shown in Figure 4B, SOX9 knockdown significantly increased AR mRNA expression.

However, relatively few studies have focused on the ability of AR to regulate SOX9 expression and activity. While the relationship of AR and SOX9 expression has not previously been examined by immunostaining in mouse and human testes, the relationship of AR and AMH has been well documented in human and mouse testes. Boukari et al. demonstrated that the lack of AR expression in Sertoli cells accounts for the absence of AMH repression during early human testicular development [12]. Similarly, immunohistochemical staining revealed high AMH expression in male mice lacking AR in their Sertoli cells [4], and human patients with androgen insensitivity syndrome (AIS) showed abnormally high levels of AMH [10,12]. Consistent with previous reports [2,10,12,31,38-41], we herein found an inverse relationship between AR and AMH, suggesting that AR is a major player in suppressing AMH expression through down-regulation of SOX9. We had done the ChIP assay to confirm that AR directly binds to putative androgen response elements in the SOX9 promoter which has been included in our another study. Taken together, these studies imply that high level of AMH driven by SOX9 could explain a physiological Sertoli cell lacking in AR during fetal and early postnatal life, which may serve to protect the testis from precocious Sertoli cell maturation, resulting in proliferation arrest and spermatogenic development. Furthermore, our results suggest that SOX9-induced AMH overexpression could also explain why pathological Sertoli cells show AR deficiencies during in adults with SCOS. This may reflect that the testes have retained precocious Sertoli cells that failed to mature, resulting in defective spermatogenesis.

Although the persistence of high AMH expression in Sertoli cells or high serum concentration in adulthood may indicate a failure in Sertoli cell maturation, it may also reflect deficiencies in the action of androgen. Thus, the clinical applications of AMH measurement in predicting quantitative and qualitative aspects of male infertility remain controversial. Although we only see limited evidence from meta-analysis, serum AMH does not appear to have diagnostic value as a stand-alone marker of persistent spermatogenesis in men with non-obstructive azoospermia [42]. AMH is secreted bi-directionally by Sertoli cells: apically into the seminiferous tubules, and basally toward the interstitium and blood circulation. Thus, AMH may be measured in both serum and seminal fluid. Serum concentrations of AMH are high until puberty, when they decrease dramatically to their low adulthood level [11]. After puberty, AMH is secreted preferentially by the apical pole of the Sertoli cell, so there are higher concentrations in seminal plasma compared to serum [42]. We herein showed that AMH is strongly expressed and locally retained in the Sertoli cell cytoplasm of testiculopathic testes (Figure 1) but not in the serum of patients (data not shown). As shown in Figure 1, these testiculopathic testes have thickened and hyalinized basal membranes that may prevent AMH from crossing the blood-testis barrier and entering circulation, perhaps explaining why serum AMH may not be a suitable prognostic marker in patients with non-obstructive azoospermia.

The etiology of SCOS is obscure, and the mechanisms underlying its pathological process are currently unknown. The proposed explanations include abnormal development with failure of the primordial germ cells to migrate into the future gonads, and secondary destruction of the germinal epithelial layer [25,26]. The term ‘partial SCOS’ is often used when a variable proportion of tubules contain no germ cells while other regions show some germ cell development, even to the elongated spermatid stage [25,26]. This is a relatively common pattern in idiopathic spermatogenic failure, suggesting that SCOS could represent a spectrum of testicular diseases with defective Sertoli cells. It also suggests that partial SCOS patients may gradually lose germs cells and progress to a complete SCOS phenotype with azoospermia. As another example, Klinefelter’s syndrome is a time-dependent degenerative disease characterized by SCO and Leydig cell hyperplasia. For these patients, it was recently suggested that clinicians should search for and cryopreserve spermatozoa in adolescents instead of adult patients [43]. While most idiopathic oligo/azoospermic patients are not diagnosed with hypogonadism, and testosterone treatment has not always effective in men with idiopathic oligo/azoospermia [44], our SCOS patients without androgen receptor gene mutations (data not shown) and presented with relatively low serum testosterone levels. Decreased circulating Testosterone in SCOS patients might be due to high AMH which has been previously reported to affect steroidogenesis by leydig cells [45,46]. It is possible that non-invasive medical treatments, such as androgen supplementation, could benefit partial SCOS patients who have suffered repeated sperm retrieval failure history, low serum testosterone levels and high AMH levels in seminal fluid, or high levels of SOX9 expression found on a needle biopsy of the testes.

In sum, we herein report a previously unidentified relationship between the down-regulation of androgen/AR signaling and SCOS, accompanied with high SOX9 and AMH expression in Sertoli cells. Our study highlights the importance of SOX9 signaling in promoting AMH expression to keep Sertoli cells in an immature state in the presence of low androgen levels. The SOX9-induced up-regulation of AMH in Sertoli cells may explain the immaturity of Sertoli cells in SCOS patients with low serum testosterone levels. Our results collectively demonstrate a novel role of SOX9 and the regulation of SOX9/AMH by AR signaling in the pathogenesis of SCOS, potentially offering a new direction in the search for androgen therapies in partial SCOS patients who have suffered repeated sperm retrieval failure history.

## Supporting Information

Figure S1 **Relative quantification of AR, SOX9, AMH mRNA levels was performed using average of 18S and GAPDH as internal controls using REST 2009 Software in adult human testes and mouse testes.** A significant downregulation of mRNA expression for AR- and up-regulation of mRNA for SOX9, AMH was seen in SCOS testes compared with normal controls (**A**). The similar results were seen in AR-knockout mice compared to controls (**B**).
(TIFF)

Figure S2 **The AR and SOX9 expression levels were measured in TM4 cells under conditions in the presence of 1x10^-8^ M DHT or 10µM flutamide, an androgen antagonis**t **for 24 hours**. Concomitant detection of β-actin mRNA in the real-time RT-PCR reaction served as a reference for relative quantification. All data are representative of at least three independent experiments and error bars represent ± SD. Asterisks (*) mark samples significantly different with P <0.05.
(TIFF)

Table S1 **Oligonucleotide primers used for quantitative real-time reverse transcriptase-polymerase chain reaction.**
(DOC)
