# Supplementary figures and images for: Up-Regulation of SOX9 in Sertoli Cells from Testiculopathic Patients Accounts for Increasing Anti-Mullerian Hormone Expression via Impaired Androgen Receptor Signaling
Source: PLoS One. 2013 Oct 1;8(10):e76303. doi: 10.1371/journal.pone.0076303 (PMC3788123; doi:10.1371/journal.pone.0076303)

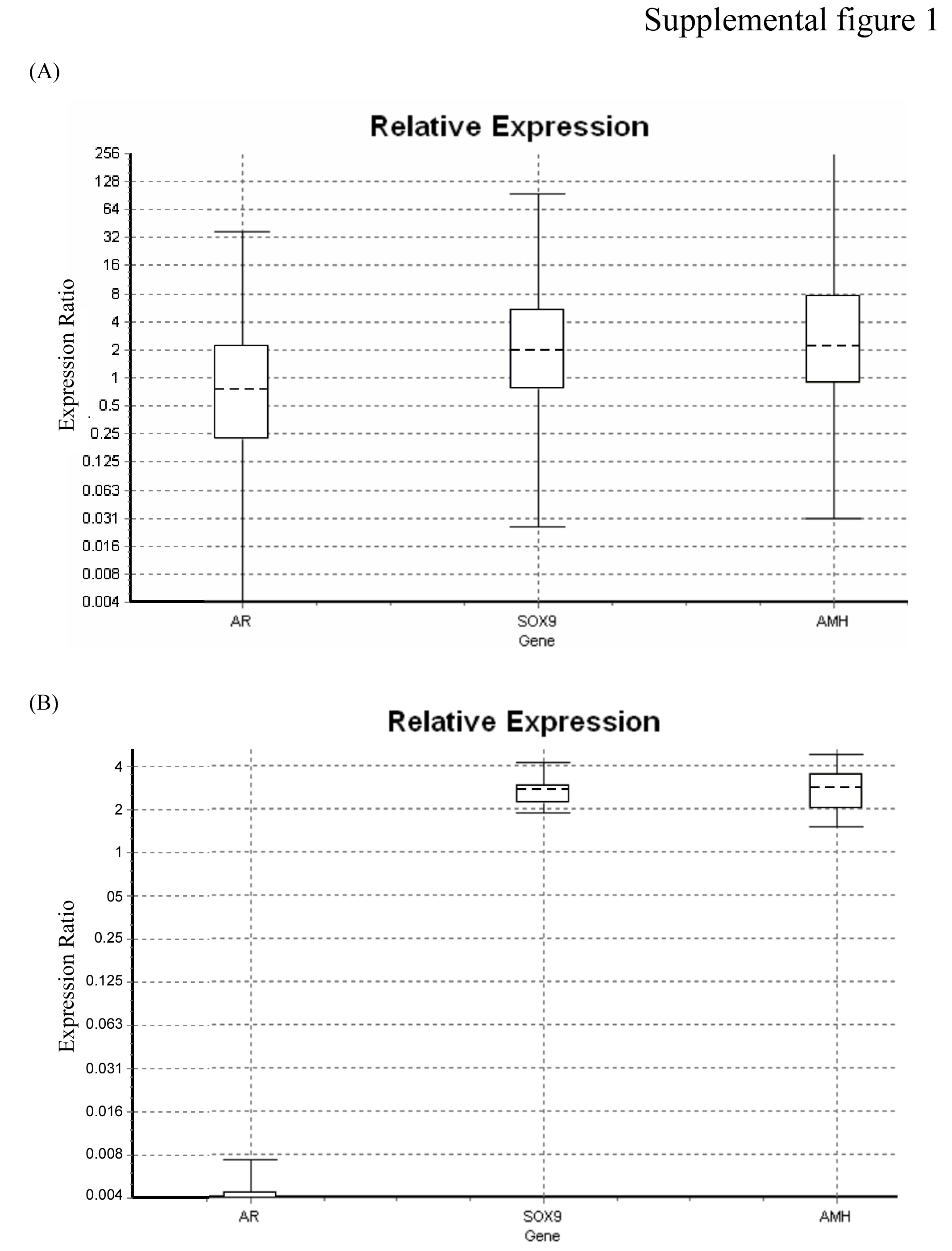

Supplement: Figure S1 — Relative quantification of AR, SOX9, AMH mRNA levels was performed using average of 18S and GAPDH as internal controls using REST 2009 Software in adult human testes and mouse testes. A significant downregulation of mRNA expression for AR- and up-regulation of mRNA for SOX9, AMH was seen in SCOS testes compared with normal controls (A). The similar results were seen in AR-knockout mice compared to controls (B). (TIFF) [file pone.0076303.s001.tiff]

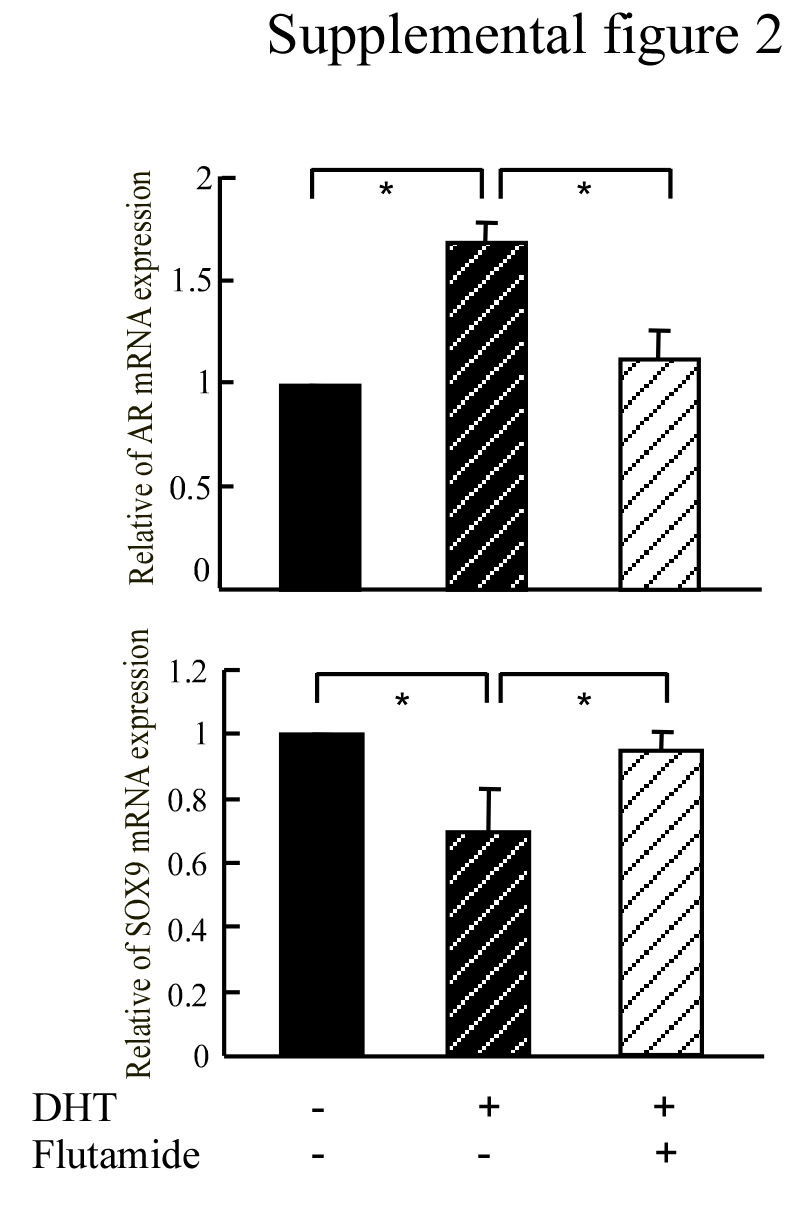

Supplement: Figure S2 — The AR and SOX9 expression levels were measured in TM4 cells under conditions in the presence of 1x10^-8^ M DHT or 10µM flutamide, an androgen antagonist for 24 hours. Concomitant detection of β-actin mRNA in the real-time RT-PCR reaction served as a reference for relative quantification. All data are representative of at least three independent experiments and error bars represent ± SD. Asterisks (*) mark samples significantly different with P <0.05. (TIFF) [file pone.0076303.s002.tiff]
