# Supplementary material for: Up-Regulation of SOX9 in Sertoli Cells from Testiculopathic Patients Accounts for Increasing Anti-Mullerian Hormone Expression via Impaired Androgen Receptor Signaling
Source: PLoS One. 2013 Oct 1;8(10):e76303. doi: 10.1371/journal.pone.0076303 (PMC3788123; doi:10.1371/journal.pone.0076303)
Supplement: Table S1 — Oligonucleotide primers used for quantitative real-time reverse transcriptase-polymerase chain reaction. (DOC) [file pone.0076303.s003.doc]

Supplemental Table 1. Oligonucleotide primers used for quantitative real-time reverse transcriptase-polymerase chain reaction.

| Gene | 5’ Primer (5’-3’) | 3’ Primer (5’-3’) |
| --- | --- | --- |
| Human | | |
| AR | TCACCGCACCTGATGTGTG | ACATGGTCCCTGGCAGTCTC |
| AMH | AGCTGTGGGCACCAGTGG | GCTCTTGTGGGCTGCCTG |
| SOX9 | GCAAAGGAGATGAAATCTGTTCTG | AAGGTTAACTGCTGGTGTTCTGAGA |
| GATA-4 | AGGCCTCTTGCAATGCGGA | CTGGTGGTGGCGTTGCTGG |
| Vimentin | CCTTGAACGCAAAGTGGAATC | GACATGCTGTTCCTGAATCTG |
| BMP4 | TTTATGAGGTTATGAAGCCCCCGGC | AGTTTCCCACCGTGTCACATTGTG |
| GDNF | TGAAACCAAGGAGGAACTGATTTT | GTCACTCACCAGCCTTCTATTCTG |
| SCF | GATGTTTTGCCAAGTCATTGTTGG | ACTGACTCTGGAATCTTTCTCAGG |
| β-actin | TCACCCACACTGTGCCCATCTACGA | CAGCGGAACCGCTCATTGCCAATGG |
| GAPDH | TGCACCACCAACTGCTTAGC | GGCATGGACTGTGGTCATGAG |
| 18S | GTAACCCGTTGAACCCCATT | CCATCCAATCGGTAGTAGTG |
| Mouse | | |
| AR | GCTGCCTTGTTATCTAGCCTCAA | AATGACCGCCATCTGGTCAT |
| AMH | CCCGCTATTTGGTGCTAACC | CGTGAAACAGCGGGAATCA |
| SOX9 | CGTCAACGGCTCCAGCA | TGCGCCCACACCATGA |
| β-actin | AGGCCAACCGTGAAAAGATG | TGTGGTACGACCAGAGGCATAC |
| GAPDH | GCACAGTCAAGGCCGAGAAT | GCCTTCTCCATGGTGGTGAA |
| 18S | CGCCGCTAGAGGTGAAATTCT | CGAACCTCCGACTTTCGTTCT |

Abbreviations: AR, androgen receptor; AMH, Anti-Müllerian hormone; BMP4, Bone morphogenetic Protein 4; GDNF, Glial cell line-derived neurotrophic factor; GATA-4, GATA binding protein 4; SCF, Stem cell factor.
